# Robust Compositional Analysis of Physical Activity and Sedentary Behaviour Data

**DOI:** 10.3390/ijerph15102248

**Published:** 2018-10-14

**Authors:** Nikola Štefelová, Jan Dygrýn, Karel Hron, Aleš Gába, Lukáš Rubín, Javier Palarea-Albaladejo

**Affiliations:** 1 Faculty of Science, Palacký University Olomouc, 771 11 Olomouc, Czech Republic; Nikola.Stefelova@seznam.cz (N.Š.); hronk@seznam.cz (K.H.); 2 Faculty of Physical Culture, Palacký University Olomouc, 771 11 Olomouc, Czech Republic; ales.gaba@upol.cz (A.G.); lukas.rubin@upol.cz (L.R.); 3 Biomathematics and Statistics Scotland, JCMB, The King’s Buildings, Edinburgh EH9 3FD, UK; javier.palarea@bioss.ac.uk

**Keywords:** compositional data, compositional linear regression, log-ratio methodology, pivot coordinates, physical activity

## Abstract

Although there is an increasing awareness of the suitability of using compositional data methodology in public health research, classical methods of statistical analysis have been primarily used so far. The present study aims to illustrate the potential of robust statistics to model movement behaviour using Czech adolescent data. We investigated: (1) the inter-relationship between various physical activity (PA) intensities, extended to model relationships by age; and (2) the associations between adolescents’ PA and sedentary behavior (SB) structure and obesity. These research questions were addressed using three different types of compositional regression analysis—compositional covariates, compositional response, and regression between compositional parts. Robust counterparts of classical regression methods were used to lessen the influence of possible outliers. We outlined the differences in both classical and robust methods of compositional data analysis. There was a pattern in Czech adolescents’ movement/non-movement behavior—extensive SB was related to higher amounts of light-intensity PA, and vigorous PA ratios formed the main source of potential aberrant observations; aging is associated with more SB and vigorous PA at the expense of light-intensity PA and moderate-intensity PA. The robust counterparts indicated that they might provide more stable estimates in the presence of outlying observations. The findings suggested that replacing time spent in SB with vigorous PA may be a powerful tool against adolescents’ obesity.

## 1. Introduction

High physical activity (PA) and a low amount of sedentary behavior (SB) are often associated with several benefits for children and adolescents, including positive effects on cardiovascular, musculoskeletal and metabolic health [1,2], well-being [3], and obesity [4]. Movement/non-movement behavior patterns in youth may extend to adulthood [5,6] and, therefore, understanding the optimal structure of time spent in various activities in early life is desirable. Current PA guidelines for adolescents around the world focus mainly on time spent in moderate PA (MPA) and vigorous PA (VPA) and encourage children and adolescents to engage in at least 60 min of moderate-to-vigorous PA every day [2]. However, being active for an hour a day does not mean that the rest of the day has no effect on overall health. Thus, some of the health benefits of meeting the PA recommendations might be lost if children are subject to extensive sitting. SB and light-intensity PA (LIPA) have not been a preferred subject of interest in public health research for decades. It was assumed that if MPA and VPA increase, SB inherently decrease. However, MPA and VPA usually account for less than 5% of the day, whilst the time spent in LIPA is approximately three times greater [7]. LIPA may also be beneficial for children’s health [8]. Additionally, excessive SB and inadequate sleep duration also represent critical health challenges to children and youth today [9]. These behaviors account for a large proportion of the day (~80%) and are considered independent risk factors for obesity [10].

The main issues in research on the relationship between movement behavior patterns and health outcomes are: (1) length of time needed for manifestation of diseases or disorders/health problems caused by an unhealthy lifestyle [11] and (2) limited possibilities to distinguish between the effects of all components of PA and SB in the prevention of health problems (e.g., obesity) [12]. It is widely thought that a greater understanding of the role of each PA and SB component with respect to other components in this age group is necessary and should be assessed in a more holistic way. Moreover, PA and SB data are compositional in nature and, hence, any proportional increase in one behavior necessarily causes a proportional decrease in one or more remaining behaviors; these behaviors are perfectly collinear if an exhaustive description of the movement behavior composition (including sleep) is provided. However, even if this is not the case, the information contained in the movement behavior composition is relative and thus scale invariant; i.e., it can be normalized to any sum (such as 1 in the case of proportions or 100 for percentages) without loss of information.

Standard statistical techniques such as multiple regression analysis are not well suited to describe the relationship between a response of interest and explanatory covariates of a compositional nature. They are not designed to accommodate and account for the relative contributions of the parts of a whole (herein “total wake time”), hereafter called compositional data [13]. Although early studies point toward the use of compositional data analysis (CODA) in PA and SB research [14,15], it was not until the work by Chastin et al. [12] that the CODA approach to PA research was introduced and discussed in a comprehensive and statistically-principled means using the latest methodological developments in the area to deal with behavioral data that represent parts of a whole (i.e., data carrying relative information), useful when either all parts or just some of them have been measured. Over the last few years, the CODA approach has notably increased its popularity in the physical activity community, and there has been a large number of studies examining the relationship between health and movement/non-movement behavior pattern among youth using CODA [16,17,18,19,20,21]. Finally, a theoretical framework, which describes the relationship between all movement/non-movement behaviors and health outcomes, has recently been updated to The Framework for Viable Integrative Research in Time-Use Epidemiology (VIRTUE framework) [22]. Up to now, primarily classical statistical analysis methods were used in PA and SB research; therefore, the aims of this study are: (1) to present a concise regression framework within CODA in the presence of outlying observations, which often occur in most real-world data sets, encouraging the use of robust statistical methods instead of their classical counterparts in these situations; and (2) to investigate the combined effect of time spent in LIPA, MPA, VPA and SB on the risk of obesity within a robust CODA framework.

## 2. Materials and Methods

### 2.1. Design of Study

Cross-sectional data from “Multifactorial research on built environment, active lifestyle and physical fitness in Czech adolescents” were used [23]. Adolescents were recruited from schools in Olomouc and Hradec Králové. To ensure a representative sample, we selected schools from different built environments and socioeconomic neighborhoods. The participants were healthy 420 adolescents (169 boys and 251 girls), whose ages ranged from 11.8–18.9 years (M = 15.1 ± 2.0), height was 167.8 ± 9.2 cm, weight was 60.7 ± 13.2 kg and BMI was 21.4 ± 3.6 kg∙m^−2^. The data were collected during the 2013–2016 academic years in elementary and secondary schools. SB, LIPA, MPA, and VPA were measured by the ActiGraph GT3X accelerometer (ActiGraph, LLC, Pensacola, FL, USA); sleep was not considered. Participants were asked to wear accelerometers for seven consecutive days, except during water-based activities. The days on which the accelerometer was worn for at least 10 h were considered valid and participants were included if at least three valid weekdays and one valid weekend day of their activity were available. The time sampling interval was set to 60 s, which has been the most frequently used epoch in the literature [24]. Each minute epoch was classified using standard counts per minute thresholds as SB (0–100 counts/min), LIPA (101–2295 counts/min), MPA (≥2296–4011 counts/min) and VPA (≥4012 counts/min), based on Evenson’s cut-points [25]. These cut-points demonstrated significant higher accuracy for SB and different levels of PA intensity than other cut-points in adolescents [26]. For the purpose of the study, averaged results of movement behavior for each participant were taken.

Body Mass Index (BMI) was calculated from the self-reported height and weight. Age and sex-adjusted BMI (zBMI) was used as an indicator of overweight and obesity. Due to the lack of individuals in each weight category, only two groups were defined; underweight/normal (<1.0 SD) and overweight/obese (≥1.0 SD). The two groups included $n_{1}$ = 344 underweight/normal and $n_{2}$ = 76 overweight/obese adolescents. The calculation was done according to WHO guideline [27].

### 2.2. Compositional Data

Compositional data (compositions) stand for all kinds of data representing parts of a whole. A vector $x=\left(x_{1},x_{2},\ldots ,\;x_{D}\right)$ is called a D-part composition when all its components (parts) are strictly positive real numbers and carry relative information [13,28]. This means that all relative information about a certain composition is contained in the ratios between its parts.

Compositional data are scale invariant, that is, if a composition is multiplied by a positive number, it does not change the ratios between the components. Accordingly, compositions can be represented without loss of information as vectors with an arbitrary constant sum constraint (typically 100 in percentage units), but the sums of components can also vary across the data set and compositional methods would still produce the same results. The operation of rescaling the initial vector so that the components add up to a constant K is formally called a closure. The resulting sample space of such constrained representation is a simplex, which is a (D−1)-dimensional subset of the ordinary real space.

Geometrically, compositions follow the Aitchison geometry on the simplex [19]. Since standard statistical methods rely on Euclidean geometry in real space, it is desirable to map compositions from the simplex into real space to be able to apply a standard statistical analysis. This can be achieved by the log-ratio methodology, which leads to the construction of coordinates that preserve the relative scale property of compositions. In the log-ratio methodology, the ratios between parts as bearers of the elemental information are replaced by log-ratios that conveniently symmetrize the interpretation of the ratios and are mathematically easier to handle. Among the different types of log-ratio transformations, the ilr (isometric log-ratio) transformations are preferred (for geometrical reasons they are referred to as ilr coordinates), as they allow to express compositions in an orthonormal coordinate system [28].

There are many ways to construct ilr coordinates but their interpretation might be quite difficult. Therefore, it is appealing to choose an orthonormal coordinate system that highlights the role of a single compositional part against the others, which have been recently called pivot coordinates [29,30]. They are constructed as follows. For a *D*−part composition x, a real vector $z=\left(z_{1},z_{2},\ldots ,\;z_{D-1}\right)$ is obtained with D−1 ilr coordinates, where

(1) $$z_{j}=\sqrt{\frac{D-j}{D-j+1}}\ln\frac{x_{j}}{\sqrt[{D-j}]{\prod_{k=j+1}^{D}x_{k}}},\;j=1,\ldots ,\;D-1.$$

Thus, all relative information about $x_{1}$ (with respect to the average contribution of the remaining parts) is explained by $z_{1}$. As an alternative interpretation, note that this first coordinate represents an aggregation of all pair-wise log-ratios of a specific compositional part to all the others (up to a normalizing constant).

By permuting the parts in the original composition, so that a different part is put in the first position each time, we can obtain D different orthonormal coordinate systems, each of them highlighting the role of one of the compositional parts. Denoting $x^{\left(l\right)}=\left(x_{l},x_{1},\ldots ,\;x_{l-1},x_{l+1},\ldots x_{D}\right)=\left(x_{1}^{\left(l\right)},x_{2}^{\left(l\right)},\ldots ,\;x_{D}^{\left(l\right)}\right),l=1,\ldots ,\;D$, produces

(2) $$z_{j}^{\left(l\right)}=\sqrt{\frac{D-j}{D-j+1}}\ln\frac{x_{j}^{\left(l\right)}}{\sqrt[{D-j}]{\prod_{k=j+1}^{D}x_{k}^{\left(l\right)}}},\;j=1,\ldots ,\;D-1,\;l=1,\ldots ,\;D.$$

Therefore, all relative information about any part $x_{l}$ is contained in $z_{1}^{\left(l\right)},\;l=1,\ldots ,\;D$ [29]. Note that different ilr coordinate systems are just a rotation of each other. This is a useful property in statistical analysis; for example, for robust estimation as well as for regression analysis, because it enables the use of an arbitrary choice of ilr coordinates to obtain the required (unique) output.

### 2.3. Compositional Regression

The objective of the regression analysis is to study the relationship between response variable(s) and explanatory variable(s). The compositional data framework has three basic regression problems. These relate to the relation between the real-valued response and compositional covariates, compositional response and real covariates, or between compositional parts themselves. The choice of pivot coordinates is convenient in all these cases, as it enhances the interpretability of the regression coefficients.

Firstly, in the situation concerning real response *Y* and compositional covariates $\left(x_{1},x_{2},\ldots ,\;x_{D}\right),$ the D regression models are considered:

(3) $$Y=\beta_{0}+\beta_{1}^{\left(l\right)}z_{1}^{\left(l\right)}+\ldots +\beta_{D-1}^{\left(l\right)}z_{D-1}^{\left(l\right)}+\varepsilon^{\left(l\right)},\;l=1,\ldots ,\;D,$$

where $z_{1}^{\left(l\right)},z_{2}^{\left(l\right)},\ldots ,\;z_{D-1}^{\left(l\right)}$ are pivot coordinates (2), $\beta_{0},\;\beta_{1}^{\left(l\right)},\;\beta_{2}^{\left(l\right)},\;\ldots ,\;\beta_{D-1}^{\left(l\right)}$ are unknown regression coefficients and $\varepsilon^{\left(l\right)}$ is the random error term in the lth model, l=1,…, D. Due to the orthogonality of different coordinate systems, the intercept term $\beta_{0}$ (as well as the model fit measures) is the same for each model. As $z_{1}^{\left(l\right)}$ explains all the relative information about part $x_{l}$, the coefficient $\beta_{1}^{\left(l\right)}$ accounts for the contribution of the dominance of this part within the given composition. Therefore, the estimate ${\hat{\beta }}_{1}^{\left(l\right)}$ is extracted from each model and the vector $\left({\hat{\beta }}_{1}^{\left(1\right)},\;{\hat{\beta }}_{1}^{\left(2\right)},\ldots ,\;{\hat{\beta }}_{1}^{\left(D\right)}\right)$ is used for interpretation purposes [31,32].

Secondly, compositional response $\left(Y_{1},\;Y_{2},\ldots ,\;Y_{D}\right)$ and real explanatory variables $\left(x_{1},x_{2},\ldots ,\;x_{p}\right)$ are considered. By expressing the composition in pivot coordinates (2), the D multiple regression models are set, where only the first coordinate $Z_{1}^{\left(l\right)}$ is of interest:

(4) $$Z_{1}^{\left(l\right)}=\beta_{0}^{\left(l\right)}+\beta_{1}^{\left(l\right)}x_{1}+\ldots +\beta_{p}^{\left(l\right)}x_{p}+\varepsilon^{\left(l\right)},\;l=1,\ldots ,\;D.$$

$\beta_{0}^{\left(l\right)},\;\beta_{1}^{\left(l\right)},\ldots ,\;\beta_{p}^{\left(l\right)}$ in (4) are unknown regression coefficients and $\varepsilon^{\left(l\right)}$ is the random error term in the lth model, l=1,…, D [33]; note, however, that these coefficients are not the same as in model (3). In both models above, it is assumed that just the response is measured with errors. Therefore, the standard regression methodology can be used for statistical inference, particularly for hypotheses testing.

Finally, for the case of regression of one compositional part on other parts, the use of orthogonal regression is appropriate, since both the response and explanatory variables are measured with errors. Accordingly, statistical inference is obtained by bootstrap [34]. Specifically, if the relationship between the dominance of part $x_{l}$ within the composition and the remaining subcomposition $\left(x_{1},x_{l-1},x_{l+1},\ldots ,\;x_{D}\right)$ is of interest, D−1 models:

(5) $$z_{1}^{\left(l\right)}=\beta_{1}^{\left(lk\right)}+\beta_{2}^{\left(lk\right)}z_{2}^{\left(lk\right)}+\ldots +\beta_{D-1}^{\left(lk\right)}z_{D-1}^{\left(lk\right)}+\varepsilon^{\left(l\right)},\;l=1,\ldots ,\;D,\;k=1,\ldots ,l-1,\;l+1,\ldots ,\;D,$$

are considered where $\beta_{1}^{\left(lk\right)},\;\beta_{2}^{\left(lk\right)},\ldots ,\;\beta_{D-1}^{\left(lk\right)}$ are unknown regression coefficients, ε is the random error term and $z_{1}^{\left(l\right)},z_{2}^{\left(lk\right)},\ldots ,\;z_{D-1}^{\left(lk\right)}$ stand for pivot coordinates (2) constructed for composition with $x_{l}$ in the first and $x_{k}$ in the second position. Then $z_{2}^{\left(lk\right)}$ contains all the relative information about $x_{k}$ with respect to the average contribution of the remaining parts of the subcomposition. Accordingly, our focus is on the interpretation of the estimate ${\hat{\beta }}_{2}^{\left(lk\right)}$ from each model, k=1,…,l−1, l+1,…, D [33], because it stands for a positive or negative influence of dominating $x_{k}$ within the subcomposition ($z_{2}^{\left(lk\right)}$) on dominance of $x_{l}$ within the parent composition (as conveyed by $z_{1}^{\left(l\right)}$).

In addition, the coefficient of determination (R^2^) was computed for all regression models to evaluate the goodness of fit. Since the main purpose of the modelling was to investigate the sign and size of the relationships, we focused on the statistical significance of the model parameters and allowed for small values of R^2^ as generally accepted in our field [35]. Note that the coefficient of determination is invariant to orthogonal coordinate representation of predictors, so for any pivot coordinates the resulting value is identical.

### 2.4. Dealing with Outliers

When it is needed to reduce the influence of possible outliers in the dataset (and thus to focus on the main trend in the dataset), robust estimation of regression coefficients [36] should be used. As all three regression models above are formulated in real coordinates, it is possible to use standard robust regression techniques. Thus, in the first two cases (models (3) and (4)), the highly efficient MM-estimation is preferable instead of the standard LS (least squares) estimation [37]. For orthogonal regression (model (5)), robust estimation based on the robust principal component analysis was applied and fast robust bootstrap is used for statistical inference [34]. In order to interpret the results from robust regression, the specific consequences of outlying observations in compositional data sets need to be taken into account. While for standard real data outliers are characterized usually by extreme value(s) in one or more variables, in the compositional case the outlyingness originates from aberrant log-ratios and, hence, irrespective of the concrete representation of the relative information conveyed by the composition. Finally, note that this also affects compositional descriptive statistics, which are represented by the centre and the variation matrix [28] and used in the present analysis to accompany the regression analysis results. A key to their robustification is the application of the robust Minimum Covariance Determinant (MCD) estimator of location and covariance [35] in any orthonormal coordinate. The MCD estimator is a highly robust estimator and transforms according to the change of coordinates (this is referred to as “affine equivariance”), so that unique results for the original compositional data are obtained.

As outlying observations occur naturally also in PA data, the use of robust methods for regression analysis in this work was selected and illustrated. The usefulness of the robust approach was demonstrated also for the case of basic descriptive statistics of compositional data such as the centre and the variation matrix [28].

## 3. Results

In the following text, the above-introduced four distinct types of activities (SB, LIPA, MPA, and VPA) were analyzed. The average daily behavior of an individual was considered representative of their habitual PA. Additionally, adolescents’ sex, age, weight and height were recorded and the latter three variables were used in the following analysis.

For the sake of succinctness, the following notation will be used:

(6) MB=(SB, LIPA, MPA, VPA)

for the entire movement behavior (MB) composition and

(7) PA=(LIPA, MPA, VPA)

for the PA subcomposition in particular.

### 3.1. Relationship between Various Movement Behaviour Intensities

At first, the authors analyzed the inter-relationships between the parts that form the MB composition. The proportional relationships were summarized in the so-called compositional variation matrix, formed by the variances of all possible pair-wise log-ratios [28] (Table 1). The lower the value of these variances, the more proportional the respective compositional parts are. Accordingly, Table 1 shows that SB and LIPA were the most proportional parts, while the least proportional pair was LIPA and VPA.

Alternatively, a robust variation matrix [38] as shown in Table 2 can be obtained by robust estimation of the covariance matrix in any pivot coordinate system (MCD estimator was used here). By comparing it with the previous (classical) variation matrix, it can be observed that the proportionality between SB and LIPA was slightly weakened, therefore now LIPA and MPA become the most proportional components.

A limitation of the variation matrix is that it does not consider the effect of other parts on the relationship between the two components involved. Moreover, it does not provide information about positive or negative directions of the relationship. To circumvent this limitation, we investigated the relationship between SB, the “non-active” part in the MB composition, and the parts in the PA sub-composition via robust orthogonal regression. For this purpose, model (5) was specified as:

(8) $$z_{1}^{\left(SB\right)}=\beta_{1}+\beta_{2}^{\left(SB,\;PA\right)}z_{2}^{\left(SB,\;PA\right)}+\beta_{3}^{\left(SB,\;PA\right)}z_{3}^{\left(SB,\;PA\right)}+\varepsilon$$

Thus, three models are obtained, where $\left(z_{1}^{\left(SB\right)},z_{2}^{\left(SB,\;PA\right)},z_{3}^{\left(SB,\;PA\right)}\right)$ are pivot coordinates for the MB composition with SB in the first position and a particular part (either LIPA, MPA or VPA) in the second position. The estimate ${\hat{\beta }}_{2}^{\left(SB,\;PA\right)}$ from each model is of interest.

The results (Table 3) imply that the relative dominance of SB with respect to the average contribution of PA parts is positively associated with the relative dominance of LIPA with respect to the average contribution of the remaining PA parts, whereas it is negatively related to the relative dominance of MPA and VPA (with respect to the average contribution of the remaining PA parts). It can be concluded that those individuals who spend a relatively large amount of time in SB also tend to spend a relatively high amount of time in LIPA and a relatively small amount of time in MPA and VPA.

### 3.2. Changes in Movement Behaviour with Increasing Age

**Figure 1:**
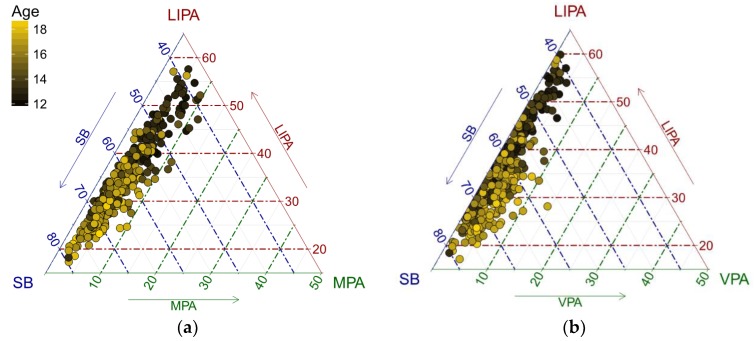

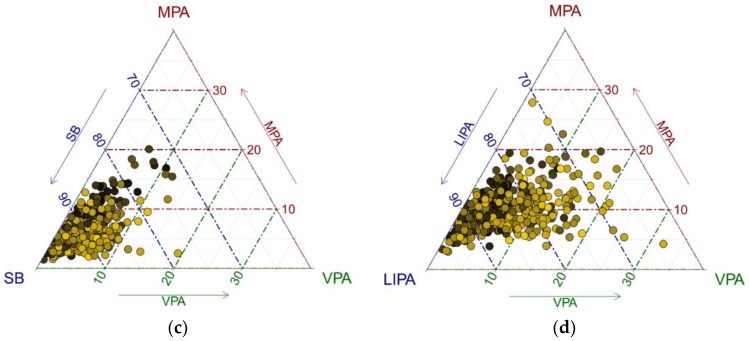
Ternary diagrams visualizing how the structure of PA and SB is associated with age. Each plot represents relationship between three of four behaviors; (**a**) SB, LIPA and MPA, (**b**) SB, LIPA and VPA, (**c**) SB, MPA and VPA, (**d**) LIPA, MPA and VPA. The lighter the point color, the higher the age of an individual. The diagrams indicate that the proportions of time spent in SB and VPA is associated with higher age, whereas the effect is the opposite for LIPA and MPA.

**Figure 2:**
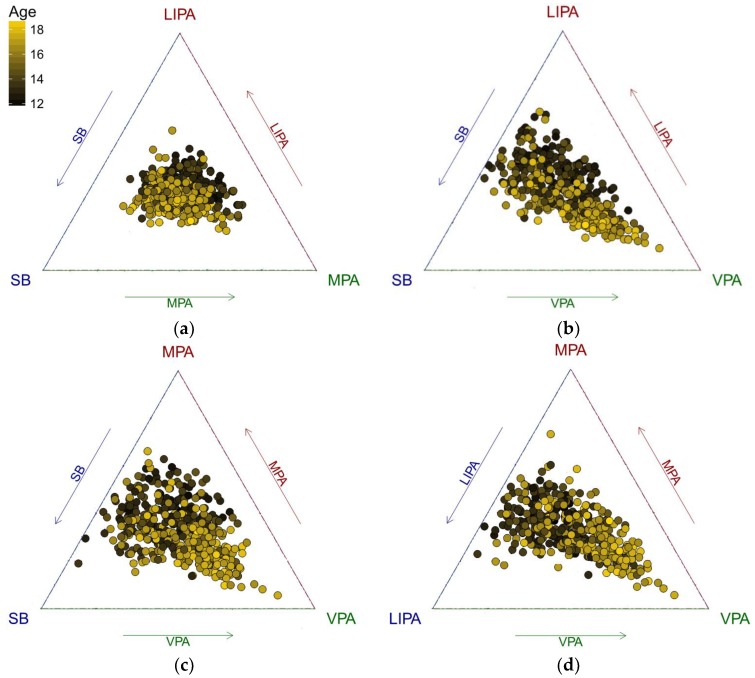
Ternary diagrams visualizing how the structure of PA is associated with age using robustly centered data. Each plot represents relationship between three of four behaviors; (**a**) SB, LIPA and MPA, (**b**) SB, LIPA and VPA, (**c**) SB, MPA and VPA, (**d**) LIPA, MPA and VPA. Plotting of the centered data is recommended when the data are concentrated near the borders of the triangle. Note that tick labels are no longer meaningful after centering.

In the following part, we investigate how age is associated with the structure of adolescents’ PA. To get an initial insight into the problem, the data were displayed in a ternary diagram, which is a standard tool for visualization of the simplex sample space for a three-part (sub)composition [28]. A color gradient was used to distinguish PA points by age (Figure 1). For a further insight, it is useful to plot centered data (Figure 2), particularly when the data are concentrated near the borders of the ternary diagram. The reason for this is the relative scale of compositional data; near the borders, the ratios between the components change substantially more than near the barycenter and this is reflected by larger distances between points in terms of the Aitchison geometry [39]. It means that near the borders, outlying observations might easily be overlooked due to the small relative values of compositional parts. Moreover, the centering operation can be robustified by computing a robust center measure, as it was the case of the present analysis, instead of the common compositional center in order to prevent possible masking of outliers. For the compositional center, using the geometric mean is a default choice in the compositional framework [28]. The robust compositional mean is then obtained by using the MCD estimator on pivot coordinates, and subsequently back-transforming, which results in a weighted geometric mean [38].

An exchange between LIPA and SB time as adolescents get older is apparent (Figure 1 and Figure 2). It is interesting to see some outliers towards VPA that might indicate intensive sport activities of adolescents. Even in the PA sub-composition, a similar trend can be observed.

The relationship between age and MB composition was further examined via a regression analysis. The MB composition was set as the response and age as the explanatory variable. Thus, the following regression model (4) was considered:

(9) $$z_{1}^{\left(MB\right)}=\beta_{0}+\beta_{1}^{\left(MB\right)}Age+\varepsilon^{\left(MB\right)}$$

Robust MM-estimation was conducted while focusing on the ${\hat{\beta }}_{1}^{\left(MB\right)}$ coefficient from each of the four models informing about increasing or decreasing dominance of the part isolated in the response pivot coordinate.

The results displayed in Table 4 support the anticipated positive association between age and the relative dominances of SB and VPA (with respect to the average contribution of the other parts). Conversely, the relative dominances of LIPA and MPA were negatively associated with age. The older they get, Czech adolescents tend to spend more time in SB and VPA at the expense of LIPA and MPA.

**Figure 3:**
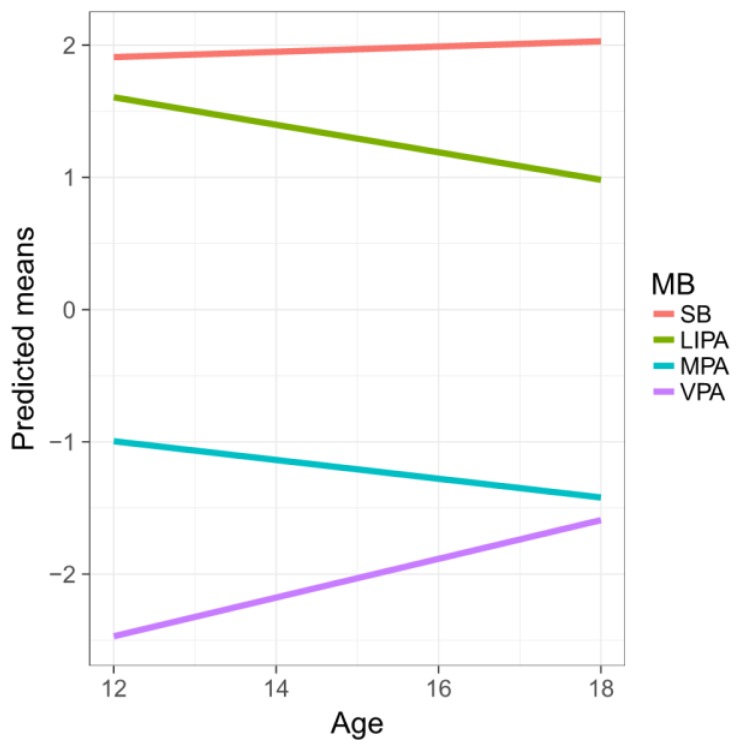
Predicted means of the dominance of the movement composition components with age. Both SB and VPA increase whereas LIPA and MPA decrease.

Figure 3 shows the predicted means for the first pivot coordinates with increasing age. Indeed, the most dramatic difference is observed for VPA, reflecting on previous observations. Note that R^2^ values differ due to the different response coordinates.

### 3.3. The Association between Movement Behaviour and Obesity 

Finally, we examined the differences in PA between adolescents from different weight groups. Table 5 shows the compositional center of MB computed for all adolescents, compared with that in the underweight/normal and overweight/obese groups.

**Figure 4:**
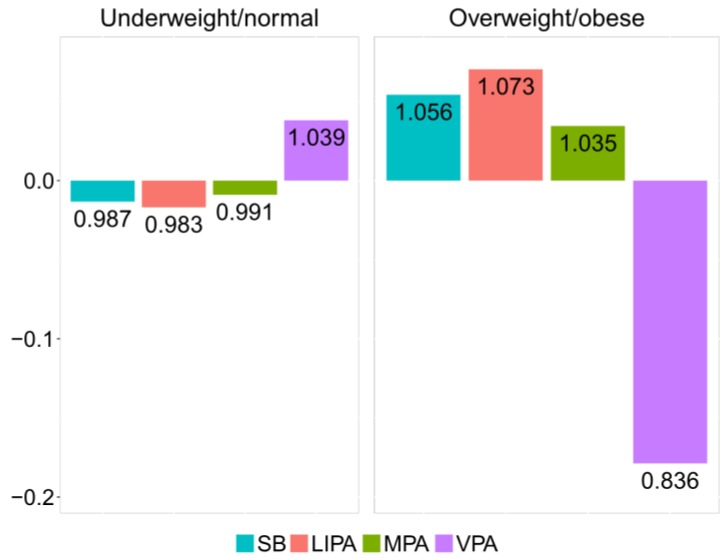
Compositional mean barplot for the underweight/normal and overweight/obese adolescent groups.

The relative difference between underweight/normal and overweight/obese groups can be visualized by the compositional mean barplot [12] (Figure 4). The vertical axis values correspond to the log-ratios between the respective group centers and the overall center after data centering. The numeric labels of the bars inform about the relative differences with respect to the overall center. Thus, in the overweight/obese group, the proportion of time spent in VPA is reduced by 16.4% relatively to the overall center. Accordingly, VPA stands out as a key driver of the difference between the underweight/normal and overweight/obese groups, stressing on the lack of VPA time in overweight/obese adolescents.

**Figure 5:**
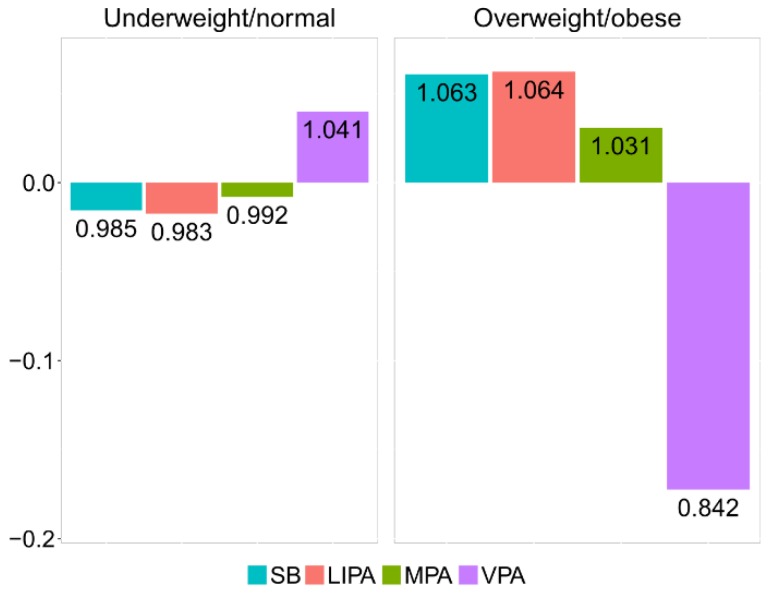
Robust compositional mean barplot for the underweight/normal and overweight/obese adolescent groups.

In order to investigate the effect of outlying observations in the whole data set, as well as in both weight groups, robust counterparts of the compositional centers and barplots were considered (Table 6 and Figure 5). Although no dramatic changes can be observed in this case, some patterns are evident. Specifically, it can be observed that the robust center for VPA was slightly higher in all groups (compared to the classical one shown in Table 5) and that the relative difference in VPA was smaller in both groups (Figure 5). This suggests the presence of some outliers (those with very low relative contribution of VPA to PA) in both groups of observations. Downplaying them thus provides a more reliable result that better summarizes adolescents’ behavior. Particularly, the robust compositional mean barplot (Figure 5) shows that the proportion of time spent in VPA in the overweight/obese group was reduced by 15.8%, relative to the overall mean composition.

The continuous character of zBMI was used to obtain more precise information about the association between PA and obesity using an appropriate regression model. zBMI was set as the response in the regression model (3) and the parts of the MB composition as covariates. Using the notation (6), the corresponding four models are as follows:

(10) $$zBMI=\beta_{0}+\beta_{1}^{\left(MB\right)}z_{1}^{\left(MB\right)}+\beta_{2}^{\left(MB\right)}z_{2}^{\left(MB\right)}+\beta_{3}^{\left(MB\right)}z_{3}^{\left(MB\right)}+\varepsilon^{\left(MB\right)},$$

where $\left(z_{1}^{\left(MB\right)},z_{2}^{\left(MB\right)},z_{3}^{\left(MB\right)}\right)$ are pivot coordinates for the MB composition resulting from sequential placement of each part originally in position l at the first position (i.e., l = 1,2,3,4 for SB, LIPA, MPA and VPA, respectively). We focused on the estimate ${\hat{\beta }}_{1}^{\left(MB\right)}$ from each of the four models, which refers to the dominance of the corresponding part within the given composition. These models were applied in the PA context in [12,40] using standard LS estimation. Here, robust MM-estimation was used instead to lessen the influence of outlying observations.

The results of MM-regression are displayed in Table 7. Accordingly, when considering just statistically significant coefficients (*p*-value < 0.05) the relative dominance of SB (with respect to the average contribution of the other parts) was in positive relationship with zBMI, while the relative dominance of VPA was in inverse relationship with zBMI. Those who spend too much time in SB and not enough time in VPA tend to have higher BMI.

**Figure 6:**
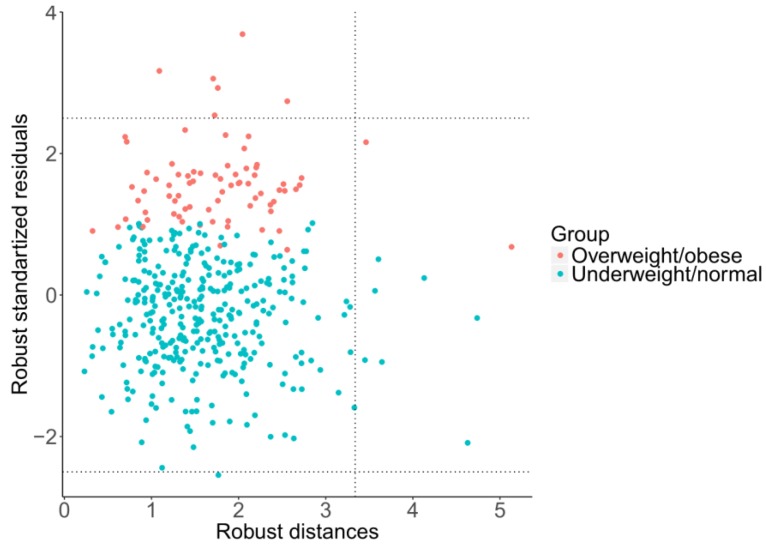
Evaluation of outlying observations from MM-regression model (10).

Moreover, using robust regression, it is possible to investigate the effects of outlying observations using a diagnostic plot of standardized residuals versus robust distances of the predictor variables [26], as shown in Figure 6. Following Rousseeuw [31], this enables us to distinguish three basic types of outliers: good leverage points, bad leverage points, and vertical outliers. Good leverage points are points (formed by response values and predictors) that are close to the regression plane; that is, good leverage points improve the precision of the regression coefficients. Bad leverage points are points that are far from the regression plane; that is, bad leverage points reduce the precision of the regression coefficients. Vertical outliers are outlying observations caused by the response, again negatively affecting the regression outputs. In this case, just one diagnostic plot (Figure 6) covers all four regression models due to mutual relationships between pivot coordinates. It can be observed that, although there are some good leverage points (middle right rectangular), bad leverage points (upper or bottom right rectangular) do not occur here. The vertical outliers (upper or bottom left rectangular) appear mostly in the overweight/obese group of adolescents, which indicates that this group has more outliers than the underweight/normal one, including some extremely obese adolescents. Despite the absence of bad leverage points, the presence of vertical outliers indicates possible differences between classical (LS) and robust regression models.

For this reason, it was sensible to perform a comparison with classical LS estimates (Table 8). MM-regression outputs (Table 7) show a stronger relationship between zBMI and relative dominance of *SB*, while the association between zBMI and relative dominance of VPA is slightly weaker (which is in line with the barplots in Figure 4 and Figure 5).

**Figure 7:**
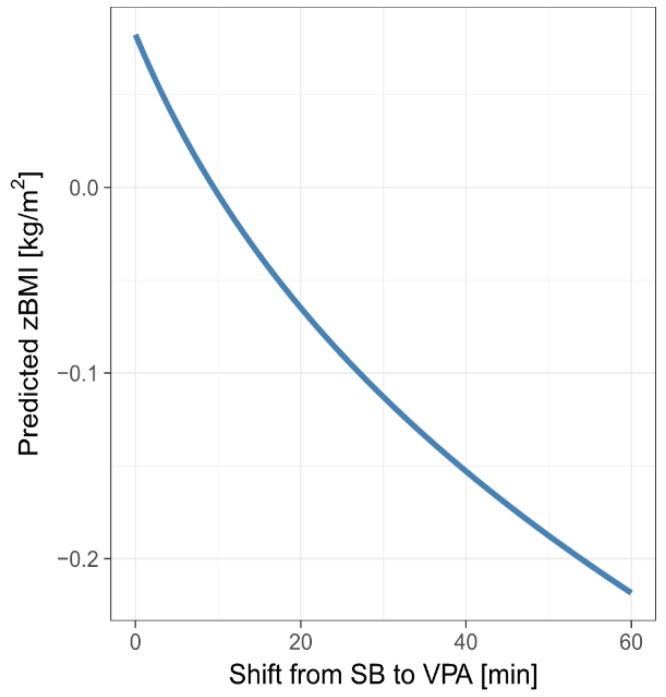
Predicted zBMI for SB-to-VPA time re-allocations between 0 and 60 min for the mean MB composition (close to 16 h).

The graph in Figure 7 shows predicted zBMI for the mean MB composition (MM-estimates are considered) when re-allocating between 0 and 60 min from SB to VPA. We considered the total MB composition to stand for 16 h, assuming 8 h of sleep a day. The predicted zBMI associated with a 60-min SB-to-VPA re-allocation was reduced by 0.30. However, due to the small value of R^2^, the predictive power of these graph is rather limited.

For example, the difference in predicted zBMI is 0.30 kg/m^2^ after re-allocating 60 min. For an average girl resp. boy from our sample (15.1 years old with a height of 1.64 m resp. 1.71 m), this would mean a 2.05 kg resp. 1.91 kg reduction in weight. For more details, see Table 9.

## 4. Discussion

This study presented a concise framework for robust CODA, mainly focusing on regression problems, in the presence of outlying observations, which often occur in real-world data sets. Although there is an increasing application of CODA methods in diverse fields of the natural [32,39,41,42,43] and social sciences [17,44,45,46,47], classical statistical inference methods have been primarily used so far. Robust CODA methods have been used almost exclusively in geochemical applications [48,49,50]. This paper demonstrates the potential of robust CODA using movement behavior data of Czech adolescents. Robust counterparts have been also introduced for common compositional descriptive statistics, including a novel way to conduct robust data centering. The key point of robust statistical methods in a compositional context is that observations with large absolute values are not necessarily those to be downplayed, but rather the focus is on aberrant log-ratios between the parts of the composition. By comparing the classical and robust variation matrices (Table 1 and Table 2), this seems to be particularly the case of pairwise log-ratios involving VPA in the present case study, which was already hinted by the ternary diagram visualization. Outliers affect classical tools such as the compositional mean barplot and ordinary LS regression; as well as the compositional centre and variation matrix used as basic descriptive statistics in CODA. The robust counterparts introduced in this work just focus on the majority of the data avoiding the extremes, hence providing more stable tools in the presence of outlying observations. They also help to reveal potential outliers, to be further analyzed and potentially suppressed. Moreover, robust centering highlights possible aberrant observations that would be masked in a classical case, where the center itself is affected by aberrant observations, particularly those close to the boundaries of the simplex. Robust regression also enabled to create a diagnostic plot to reveal vertical outliers and leverage points and enhance interpretation of the modelling; of course, similar diagnostics would also be possible for other robust regression models discussed in the manuscript. The present study demonstrated the role of orthonormal coordinates in the representation of compositional covariates, leading to the same goodness-of-fit measures and diagnostic plots for all possible choices of pivot coordinates.

The present study includes three main findings. Firstly, by using the classical compositional variation matrix, SB and LIPA showed the highest proportionality. These two parts also showed the highest proportionality (except for combination with sleep) in the study conducted by Carson et al. [16], where the same approach was applied. Using a robust compositional variation matrix, LIPA and MPA showed the highest proportionality, although SB and LIPA still retained a strong relative relationship. Moreover, both approaches have shown the highest log-ratio variances in cases where VPA was involved (reduced by using the robust approach though), indicating a possible presence of aberrant log-ratios. Although there were mostly small differences between the classical and robust variation matrices in this illustrative application (up to those variances where VPA was involved), it is well-established that the use of robust statistical methods in general tends to improve estimates in cases where an increasing presence of outliers distorts the information in the data set [36]. Secondly, an interesting feature was revealed when age was considered as an explanatory variable. Namely, with higher age the relative contributions of SB was expected to increase and VPA was expected to decrease. There is consistent evidence that SB increases with age in school-aged children and adolescents [51], but an increase in VPA with age is a rather unexpected finding. Given the higher number of outliers towards VPA (Figure 1 and Figure 2), this might be explained by intense sport activities of some adolescents pulling up the trend, even when using robust regression to quantify the relationships. The effect of VPA can be better seen in Figure 2 using centered compositions, which helps to avoid the artifacts of the relative scale of the data near the borders of the ternary diagram and facilitates the interpretation of distances between points in an (almost) usual, Euclidean, sense. Regarding the fact that in the relative scale the ratios are responsible for dissimilarities between compositions, low proportions of some components (here MPA and VPA in particular with respect to SB and LIPA, respectively; see Figure 1) might also be a source of outliers, whose impact is reduced automatically by using robust centering, instead of the classical one in Figure 2. Finally, a significant relationship between VPA and adolescents’ obesity was demonstrated. In the overweight/obese group, the relative time spent in VPA was reduced by 15.8% to the overall mean movement behavior composition, the highest reduction (using moderate-to-vigorous PA though) to overall mean composition in obese group was also found in [16].

In summary, this study has systematically considered and presented CODA regression methods which are, by construction, robust to the presence of outlying observations. They were discussed in relation to standard ‘non-robust’ regression models, as those applied in previous studies using movement behavior data [12,30,40]. Robust MM regression, which aims to retain robustness and resistance against outliers whilst gaining in statistical efficiency, is a proper tool for dealing with real-world data with natural presence of extreme observations; similar goals are also achieved by robust orthogonal regression. Accordingly, robust regression enables to extract the main trends in the data although we should be aware that from the methodological perspective, robust estimators are less efficient (precise) than the classical ones. At the same time, the associated diagnostics tools help to reveal deviating data points, which contributes to enhanced interpretation of the results. Although it is formally well-established that robust methods are generally less sensible to the presence of outlying observations, a simulation study would be necessary to determine and test for the significance of the differences in estimates between classical and robust models in an application. In our case study, we relied just on the general fact that robust estimators take into account majority of data and thus downgrade the influence of aberrant observations.

There are several strengths and limitations to this study that should be discussed. The availability of data of a relatively large number of individuals supported the statistical power of the regression significance tests and facilitated the application of robust statistical methods. Accordingly, trustworthy trends in movement behavior composition of adolescents were revealed, including those with respect to variables such as zBMI and age. The estimate of SB and PA used in this study was measured by the hip-worn ActiGraph accelerometer, the most commonly used validated accelerometer in PA research. However, due to the design of the study, the participants did not wear the accelerometer overnight, so we were not able to conduct an analysis on 24-h time budget data.

The accelerometers were set-up to record data at 60-s epochs, which can result in lower estimates of accelerometer-determined MPA and VPA [52]. The SB and PA intensities were only analyzed using Evenson cut-points. It is assumed that the decision of selecting among various cut-points significantly influences the levels of MPA and VPA [4]. Finally, BMI was calculated from self-reported height and weight. All this may introduce bias in relationship between SB, PA and obesity. Moreover, it was not possible to infer causality relationships from this observational cross-sectional study or totally discard the potential influence of unmeasured confounding variables.

## 5. Conclusions

We performed a concise robust statistical analysis of PA and SB data by using both common compositional descriptive statistics tools and robust regression modelling, which is a novel aspect of CODA modelling in physical activity research. The robust counterparts in the application case indicated that they might provide more stable estimates in the presence of outlying observations. Although there were no dramatic differences between classical and robust methods in this particular study, in a general case the impact of suppressing outlying observations might be substantial. The findings stressed that replacing time spent in SB with VPA can be a powerful tool against adolescents’ obesity. It was demonstrated that there was a pattern in Czech adolescents’ movement/non-movement behavior such that they spent more time in SB and VPA with increasing age and, consequently, less time in LIPA and MPA. Finally, it was shown that those with a greater proportion of time spent in SB also spent a considerable amount of time in LIPA and, accordingly, smaller amounts of time in MPA and VPA. It was observed that SB and VPA in particular were behaviors with high importance in the movement behavior composition in Czech adolescents, irrespective of their role as covariates or response in regression modelling, and they also drove the relative contributions of the related LIPA and MPA behaviors. Nonetheless, it could be challenging for policy makers to highlight the role of VPA and encourage such PA in the adolescents’ population.

## Figures and Tables

**Table 1:** Compositional variation matrix of adolescents’ movement behavior composition.

MB	SB	LIPA	MPA	VPA
SB	0	0.1460	0.2544	0.7757
LIPA	0.1460	0	0.1577	0.8993
MPA	0.2544	0.1577	0	0.7828
VPA	0.7757	0.8993	0.7828	0

Note: MB, movement behavior; SB, sedentary behavior; LIPA, light-intensity physical activity; MPA, moderate physical activity; VPA, vigorous physical activity.

**Table 2:** Robust compositional variation matrix of adolescents’ movement behavior composition.

MB	SB	LIPA	MPA	VPA
SB	0	0.1669	0.2801	0.6624
LIPA	0.1669	0	0.1578	0.8351
MPA	0.2801	0.1578	0	0.6946
VPA	0.6624	0.8351	0.6946	0

Note: MB, movement behaviour; SB, sedentary behaviour; LIPA, light-intensity physical activity; MPA, moderate physical activity; VPA, vigorous physical activity.

**Table 3:** Robust orthogonal regression estimates for regression model (8).

PA	${\hat{\beta }}_{2}^{\left(PA\right)}$	Standard Error	*p*-Value
LIPA	1.4295	0.1597	<0.001
MPA	−1.3179	0.1816	<0.001
VPA	−0.1116	0.0397	0.014

Note: R^2^ = 0.3005 for all models. LIPA, light-intensity physical activity; MPA, moderate physical activity; VPA, vigorous physical activity.

**Table 4:** MM-regression estimates from model (9).

MB	${\hat{\beta }}_{1}^{\left(PA\right)}$	Standard Error	*p*-Value	$R^{2}$
SB	0.0200	0.0087	0.023	0.0119
LIPA	−0.1040	0.0074	<0.001	0.2969
MPA	−0.0709	0.0088	<0.001	0.1335
VPA	0.1464	0.0157	<0.001	0.1674

Note: MB, movement behavior; SB, sedentary behavior; LIPA, light-intensity physical activity; MPA, moderate physical activity; VPA, vigorous physical activity.

**Table 5:** Center of movement behavior for the whole data set and for underweight/normal and overweight/obese subgroups.

Group	SB	LIPA	MPA	VPA
All	0.60749	0.33605	0.03812	0.01833
Underweight/normal	0.60753	0.33488	0.03829	0.01930
Overweight/obese	0.60694	0.34121	0.03734	0.01451

Note: Values expressed in proportion; MB, movement behaviour; SB, sedentary behaviour; LIPA, light-intensity physical activity; MPA, moderate physical activity; VPA, vigorous physical activity.

**Table 6:** Robust center of movement behavior for the whole data set and for underweight/normal and overweight/obese subgroups.

Group	SB	LIPA	MPA	VPA
All	0.60517	0.33675	0.03833	0.01975
Underweight/normal	0.60557	0.33550	0.03840	0.02053
Overweight/obese	0.60806	0.33885	0.03738	0.01571

Note: Values expressed in proportion. SB, sedentary behavior; LIPA, light-intensity physical activity; MPA, moderate physical activity; VPA, vigorous physical activity.

**Table 7:** MM-regression estimates for compositional model (10).

MB	${\hat{\beta }}_{1}^{\left(MB\right)}$	Standard Error	*p*-Value
SB	0.3756	0.1897	0.048
LIPA	−0.3077	0.1964	0.118
MPA	0.1388	0.1436	0.334
VPA	−0.2066	0.0813	0.011

Note: R^2^ = 0.0258 for all models. MB, movement behavior; SB, sedentary behavior; LIPA, light-intensity physical activity; MPA, moderate physical activity; VPA, vigorous physical activity.

**Table 8:** LS-regression estimates for model (10).

MB	${\hat{\beta }}_{1}^{\left(MB\right)}$	Standard Error	*p*-Value
SB	0.3237	0.1616	0.046
LIPA	−0.2277	0.1966	0.248
MPA	0.1388	0.1550	0.439
VPA	−0.2163	0.0690	0.002

Note: $R^{2}=0.0254$ for all models. MB, movement behavior; SB, sedentary behavior; LIPA, light-intensity physical activity; MPA, moderate physical activity; VPA, vigorous physical activity.

**Table 9:** Predicted decrease in zBMI/BMI when re-allocating 15/30/45/60 min from SB to VPA.

Shift from SB to VPA (min)	15	30	45	60
Predicted zBMI (kg/m^2^)	0.12	0.19	0.25	0.30
Weight reduction for an “average” girl (kg)	0.83	1.35	1.73	2.05
Weight reduction for an “average” boy (kg)	0.78	1.26	1.62	1.91

Note: SB, sedentary behavior; VPA, vigorous physical activity.
